# Global Population Genomics Links Mobilome Architecture to Antimicrobial Resistance Gene Burden in *Pseudomonas aeruginosa*

**DOI:** 10.34133/csbj.0249

**Published:** 2026-11-05

**Authors:** Sirikan Suwannasin, Ratchara Kalawong, Thitaporn Dechathai, Thunchanok Yaikhan, Sarunyou Chusri, Prasit Palittapongarnpim, Komwit Surachat

**Affiliations:** ^1^ Department of Biomedical Sciences and Biomedical Engineering, Faculty of Medicine, Prince of Songkla University, Songkhla 90110, Thailand.; ^2^ Pornchai Matangkasombut Center for Microbial Genomics, Department of Microbiology, Faculty of Science, Mahidol University, Bangkok, Thailand.; ^3^ Division of Infectious Diseases, Department of Internal Medicine, Faculty of Medicine, Prince of Songkla University, Songkhla 90110, Thailand.

## Abstract

*Pseudomonas aeruginosa* is a critical opportunistic pathogen responsible for severe healthcare-associated infections globally. Understanding its population structure and the interplay between antimicrobial resistance, mobile genetic elements (mobilome), and virulence profiles is essential for combating its spread. This study analyzes 7,341 quality-filtered *P. aeruginosa* genomes from 84 countries to define the global population structure and characterize the relationships between the mobilome, antimicrobial resistance gene (ARG) burden, and virulence profiles. Using a consensus resistome approach alongside specialized bioinformatic tools for mobile genetic elements and virulence factors, we found that the global population exhibits extensive genomic diversity alongside prominent international high-risk clones like ST235 and ST111. Acquired-candidate ARG burden was most strongly associated with integron-related features, whereas insertion sequence metrics showed moderate unadjusted correlations, with no independent association for total IS count after multivariable adjustment. Plasmid-associated metrics showed comparatively weaker associations. While virulence-associated gene content remains largely conserved across the population, type III secretion system effectors *exoU* and *exoS* display a largely reciprocal and lineage-specific distribution. Furthermore, carbapenemase genes show distinct geographic and clonal patterns, highlighting a dual dynamic of international high-risk clone dissemination and regional acquisition. These findings are consistent with a major contribution of mobile genetic elements to ARG accumulation in *P. aeruginosa*, emphasizing the need for continuous genomic surveillance to track and contain emerging strains.

## Introduction

*Pseudomonas aeruginosa* is a metabolically versatile gram-negative bacterium and a major cause of healthcare-associated and opportunistic infections, including ventilator-associated pneumonia, bloodstream infection, urinary tract infection, wound and burn infection, and chronic pulmonary disease in people with cystic fibrosis [1,2]. Its low outer-membrane permeability, inducible chromosomal beta-lactamase, multidrug efflux systems, biofilm formation, and capacity for rapid adaptive evolution make treatment particularly challenging. These features contributed to the inclusion of carbapenem-resistant *P. aeruginosa* among the World Health Organization bacterial priority pathogens [3,4]. The clinical success of *P. aeruginosa* is driven by the interplay between antimicrobial resistance (AMR) and a diverse repertoire of virulence factors [5]. Resistance arises from a combination of intrinsic mechanisms, including low outer-membrane permeability, multidrug efflux systems, and chromosomally encoded beta-lactamases, as well as acquired resistance genes introduced via horizontal gene transfer [6]. These features have facilitated the global emergence of multidrug-resistant and extensively drug-resistant lineages, particularly in hospital environments. In parallel, *P. aeruginosa* encodes a wide array of virulence determinants, including type III secretion system (T3SS) effectors, quorum-sensing regulators, biofilm-associated genes, iron acquisition systems, and secreted toxins, which collectively enable host colonization, immune evasion, and tissue damage across diverse ecological niches [7–9].

Advances in whole-genome sequencing have enabled detailed characterization of the genetic basis of resistance and virulence in *P. aeruginosa*. Previous genome-based studies have provided valuable insights into AMR gene profiles, virulence-associated loci, and population structure, particularly within regional surveillance programs or disease-specific cohorts [10]. These studies have identified globally disseminated high-risk clones, such as ST235 and ST111, and have highlighted the importance of clonal expansion in the spread of multidrug resistance. The dominant sequence types (STs) identified in this study are consistent with previously reported epidemiologically successful high-risk clones, including ST235, ST111, ST233, ST244, ST357, ST308, ST175, ST277, ST654, ST298, ST253, and ST395 [11–13]. Several frequently sampled STs overlapped with published high-risk clones; this overlap does not establish that the public-genome collection represents the circulating *P. aeruginosa* population. However, most existing genomic analyses have been limited in scale, geographically restricted, or focused on AMR and virulence separately. As a result, the global distribution of resistance and virulence determinants, and their relationships with major clonal lineages, remains incompletely understood.

Despite the extensive availability of genome sequences, important gaps remain in our understanding of AMR and virulence in *P. aeruginosa*. There is a need for integrated analyses that consider multiple hosts and ecological contexts to clarify the role of population lineage in structuring resistance and virulence traits. Additionally, the relationship between AMR burden and virulence potential on a global scale remains inadequately explored, particularly in terms of whether these traits are connected or independently acquired. Addressing these gaps is crucial for understanding the evolutionary dynamics of *P. aeruginosa*.

This study analyzed 7,341 quality-filtered *P. aeruginosa* genomes from the RefSeq NCBI database, integrating metadata with annotations of AMR genes, virulence factors, and mobile genetic elements (MGEs). We aimed to define the global population structure. We characterized associations between mobilome features and acquired-candidate antimicrobial resistance gene (ARG) burden, and compared virulence-associated profiles across lineages. This analysis describes the sampled public-genome collection and emphasizes the need for lineage-aware genomic surveillance to monitor high-risk clones.

## Materials and Methods

### Genome collection, metadata, and quality filtering

All assemblies annotated as *P. aeruginosa* were retrieved from the NCBI Assembly/Genome resources using the NCBI Datasets command-line interface on 2026 April 1. Metagenome-assembled genomes and assemblies lacking species-level taxonomic assignment were excluded. Assembly quality was evaluated with QUAST v5.2.0 [14], and genome completeness and contamination were assessed with CheckM v1.2.3 [15]. Assemblies were retained following quality criteria: number of contigs ≤100, N50 ≥100 kb with completeness >95%, and contamination <5%.

Of 15,447 publicly available assemblies, 7,341 met our quality criteria and remained after quality filtering, duplicate removal, and metadata curation for downstream analyses. Associated metadata, including accession identifiers, assembly levels, collection dates, geographic origins, hosts, and isolation sources, were retrieved from NCBI records. Missing fields were supplemented, where available, from the European Nucleotide Archive (ENA), and records were cross-checked against the Pseudomonas Genome Database (Table S1).

### Multilocus sequence typing and lineage definitions

Multilocus sequence typing (MLST) was performed with mlst v2.33.1 (Seemann T, https://github.com/tseemann/mlst) using the *P. aeruginosa* PubMLST scheme [16]. Comparisons involving high-risk clones used a prespecified literature-based ST list derived from Ref. [13]. The prespecified high-risk-clone list comprised ST235, ST111, ST233, ST244, ST357, ST308, ST175, ST277, ST654, ST298, ST253, and ST395.

### Consensus resistome analysis

AMR determinants were identified independently with AMRFinderPlus v4.0.23 [17], ResFinder [18] through ABRicate v1.0.1 (Seemann T, https://github.com/tseemann/abricate), and CARD using the Comprehensive Antibiotic Resistance Database [19]. The databases were accessed on 2026 April 20. A minimum nucleotide sequence identity of ≥80% and gene coverage of ≥80% were applied as screening thresholds. For each detected ARG, sequence identity, gene coverage, and genomic positions (start–end coordinates) were recorded separately for each detection tool (Table S2). Gene names were normalized across databases, and genomic coordinates were compared to assess locus-level agreement among detection tools. Overlapping genomic regions, coordinate concordance, and the number of supporting tools were documented to evaluate the consistency of ARG annotations. Duplicate hits corresponding to the same normalized gene designation and genomic locus were counted only once per genome. A conservative consensus resistome was constructed by retaining ARG candidates supported by at least 2 of the 3 detection tools (Table S3). For mobilome association analyses, the outcome was the count of genes designated as acquired candidates in the gene-level classification supplied in Table S2.2.

Genomes were classified as genotypically carbapenemase-positive if they carried at least one consensus gene of an acquired carbapenemase family: VIM, IMP, NDM, KPC, SPM, and GES variants with documented carbapenem-hydrolyzing activity [20–22], and other families (GIM, DIM, SIM, and FIM). GES alleles without established carbapenemase activity, including GES-1 and GES-9, were excluded [23]. Intrinsic chromosomal OXA-50-family alleles were excluded. Genomes were grouped by detected family for lineage, geographic, and temporal summaries; a genome carrying more than one family contributed to each.

### Virulence-associated gene analysis

Virulence-associated genes were identified by screening assembled genome contigs against the Virulence Factor Database (VFDB) [24] using ABRicate v1.0.1 (Seemann T, https://github.com/tseemann/abricate), with cutoff threshold ≥80% nucleotide identity and ≥80% coverage. Virulence genes were classified according to their prevalence across all genomes as core (≥99%), near-core (95% to <99%), accessory (10% to <95%), or rare (<10%).

To complement the VFDB-based analysis, exolysin A (ExlA) was independently screened using BLASTP with the ExlA protein sequence from *Pseudomonas paraeruginosa* PA7 (locus PA7_4642) as the reference. Candidate ExlA homologs were evaluated based on amino acid sequence identity and query coverage. High-similarity hits were defined using thresholds of ≥80% amino acid identity and ≥90% query coverage.

### Mobilome analysis

Integron-associated structures were identified using IntegronFinder v2.0.6 [25]. Complete integrons, In0 elements, CALIN structures, integrase genes, *attC* sites, and gene cassettes were summarized separately. A genome was considered integron-associated if at least one complete integron, In0, or CALIN structure was detected.

Insertion sequences (ISs) were identified using ISEScan v1.7.3 [26]. Complete and partial IS elements were aggregated for each genome, and both the total number of IS elements and the number of distinct IS families were recorded. MobileElementFinder v1.1.2 was used to identify reference-associated integrating MGEs, including integrative conjugative elements (ICEs) and integrative mobilizable elements [27].

Plasmid-associated contigs and predicted plasmid mobility were reconstructed using MOB-suite v3.0.0 [28]. Predicted plasmids were classified as conjugative, mobilizable, or non-mobilizable. PlasmidFinder v2.1.6 was used independently to detect known plasmid replicon sequences [29].

### Phylogenomic analysis

Genome assemblies were annotated using Prokka v1.14.6 [30], with the genus specified as *Pseudomonas* and the species as *P. aeruginosa*. The resulting GFF files were used as input to PIRATE v1.0.5 [31] for pangenome analysis. PIRATE clustering was performed using amino acid identity thresholds of 50%, 60%, 70%, 80%, 90%, 95%, and 98%, with coding sequence (CDS) features included in the analysis. Gene-family sequences were aligned using Multiple Alignment using Fast Fourier Transform (MAFFT) within the PIRATE workflow. A maximum-likelihood phylogeny was inferred from the core-gene alignment using IQ-TREE2 v2.3.6 [32] under the general time-reversible nucleotide substitution model with 1,000 ultrafast bootstrap replicates. No recombination masking was applied before tree reconstruction. The tree was treated as unrooted, with no outgroup-based biological rooting applied. The resulting phylogeny was initially explored in iTOL v7.5 [33]; the final figure was drawn with custom Python scripts, using midpoint rooting for display only, and annotated with STs, T3SS effector profiles, acquired-candidate ARG burden, carbapenemase gene families, and *exlA* status. Groups 1 to 3 were defined by the 2 deepest splits of the midpoint-rooted tree.

### Statistical analysis and reproducibility

Continuous variables were summarized using medians and interquartile ranges (IQRs), and binary variables were summarized as counts and percentages. Exploratory associations between ARG count and mobilome features were assessed using Spearman’s rank correlation. ARG counts were compared between MGE-positive and MGE-negative groups using the Mann–Whitney *U* test. Exploratory associations between acquired-candidate ARG burden and 12 prespecified mobilome features were assessed using Spearman’s rank correlation. The resulting 12 *P* values were treated as a single family of multiple hypothesis tests and adjusted for false discovery rate using the Benjamini–Hochberg procedure. Adjusted *P* values are reported as *q* values. The predefined Mann–Whitney comparison between integron-positive and integron-negative genomes and the multivariable GEE model were not subjected to Benjamini–Hochberg correction.

ARG count was modeled using a Poisson generalized estimating equation with an exchangeable working-correlation structure. ST was used as the clustering variable, and country group was included as a fixed-effect covariate. IS and MobileElementFinder counts were log-transformed and standardized before analysis. Model estimates are reported as adjusted rate ratios (aRRs) with 95% confidence intervals (CIs). Overdispersion and multicollinearity were assessed using the Pearson dispersion statistic and variance inflation factors, respectively.

For temporal summaries, each genome was assigned the first calendar year of its recorded collection date or date range. Annual proportions of carbapenemase-positive genomes (2000 to 2024) are reported with 95% Wilson CIs, and a genome-level logistic regression of carbapenemase carriage on collection year was used as a descriptive trend; a sensitivity analysis restricted to years with at least 30 genomes gave the same estimate. Associations between mean ARG and virulence-gene burdens across STs with at least 20 genomes were assessed with Spearman’s rank correlation. All figures, including the phylogeny, were generated from the supplementary tables and the IQ-TREE2 tree with Python scripts (matplotlib) that are provided with this submission.

## Results

### Dataset overview and population structure

Of 15,447 downloaded *P. aeruginosa* genome assemblies, 7,341 were retained after quality filtering, duplicate removal, and dataset curation. The curated collection represented 84 countries. Collection-year information was available for 6,230 genomes (84.9%), including 3,838 collected between 2016 and 2024. Human-associated isolates predominated (*n* = 5,712; 77.8%), followed by environmental (*n* = 617; 8.4%), animal-associated (*n* = 224; 3.1%), and food-associated (*n* = 58; 0.8%) isolates, while 730 genomes (9.9%) had unknown source-type assignments.

Contig-level assemblies were most common (*n* = 4,417; 60.2%), followed by scaffold assemblies (*n* = 1,408; 19.2%), complete genomes (*n* = 1,367; 18.6%), and chromosome-level assemblies (*n* = 149; 2.0%). MLST assigned 6,951 genomes (94.7%) to 1,169 distinct STs, whereas 390 genomes (5.3%) remained untyped. The most frequent STs were ST235 (*n* = 276), ST244 (*n* = 265), ST253 (*n* = 218), ST274 (*n* = 160), ST155 (*n* = 153), ST17 (*n* = 141), ST111 (*n* = 124), ST233 (*n* = 120), ST395 (*n* = 116), and ST146 (*n* = 112). Several of these STs, including ST235, ST244, and ST111, have been associated with internationally disseminated, epidemiologically important lineages (Fig. 1).

**Fig. 1.**
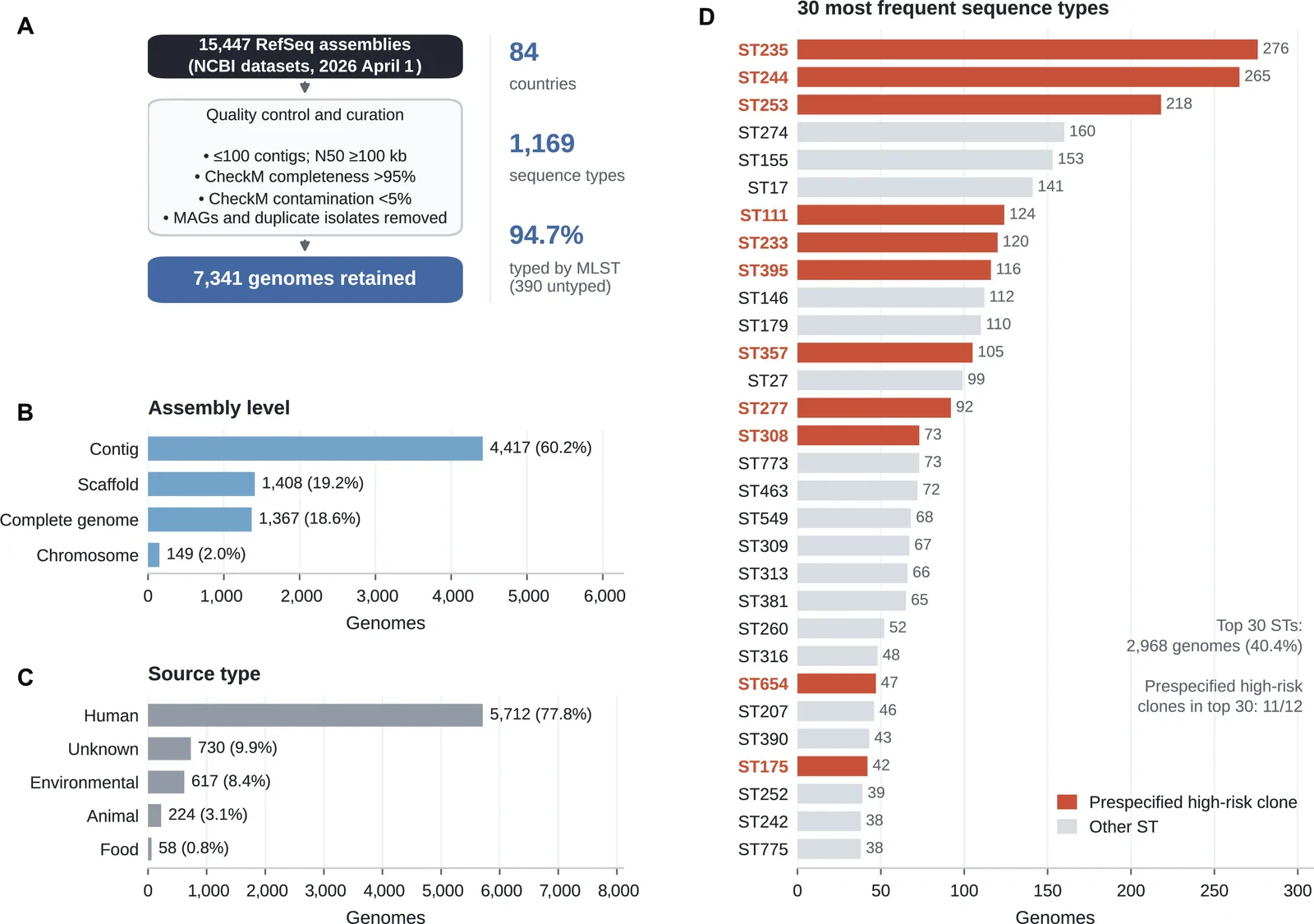
Dataset overview and population structure. (A) Workflow for genome retrieval, quality filtering, and curation, with summary characteristics of the 7,341 retained genomes. (B) Assembly level and (C) curated source type of the retained genomes; labels give counts and percentages. (D) The 30 most frequent multilocus sequence types (STs), comprising 2,968 genomes (40.4%). Prespecified high-risk clones (see the “Multilocus sequence typing and lineage definitions” section) are shown in red; ST298 is not among the 30 most frequent STs.

### Consensus resistome and lineage-specific resistance profiles

The consensus analysis identified 554 nonredundant ARGs supported by at least 2 of AMRFinderPlus, ResFinder, and CARD (Tables S2 and S3). Each gene was assigned to a category (Table S2.2): intrinsic (192 gene/allele designations; chromosomally encoded species-wide determinants), acquired candidate (255), requires verification (102; mainly *bla*_OXA_ alleles whose origin could not be assigned from the gene name alone), non-antibiotic marker (4; *qac* genes), and context-dependent (1; *crpP*). Individual genomes carried 5 to 32 consensus ARGs (median 9; IQR 8 to 9). Intrinsic genes formed a near-constant baseline (median 7 per genome; range 4 to 9), whereas acquired-candidate genes were detected in 24.2% of genomes (median 0; range 0 to 21).

The most prevalent consensus ARGs were all intrinsic: *aph(3′)-IIb* (aminoglycoside; 7,333 genomes; 99.9%), *fosA* (fosfomycin; 7,329; 99.8%), *mexX* (aminoglycoside/multidrug efflux; 7,279; 99.2%), *mexE* (multidrug efflux; 7,265; 99.0%), *mexA* (multidrug efflux; 7,244; 98.7%), and *catB7* (phenicol; 7,231; 98.5%). Among genes associated with mobile elements, *crpP* (fluoroquinolone resistance; context-dependent) was detected in 4,094 genomes (55.8%), *sul1* (sulfonamide; acquired candidate) was detected in 1,290 (17.6%), and the quaternary ammonium compound marker *qacEΔ1* (non-antibiotic marker) was detected in 772 (10.5%).

Acquired carbapenemase genes (β-lactam class) showed marked lineage specificity: *bla*_VIM-2_ was detected in 275 genomes (3.7%), most frequently in ST233 (65.0% of genomes), ST111 (44.4%), and ST823 (all 18 genomes); *bla*_NDM-1_ was detected in 159 genomes (2.2%), mainly in ST773 (69.9%), ST654 (53.2%), and ST308 (34.2%); and *bla*_KPC-2_ was detected in 160 genomes (2.2%), most frequently in ST463 (70.8%), ST1212 (64.3%), and ST485 (50.0%).

The distribution of consensus ARGs across the 30 most prevalent STs is shown in Fig. 2. Intrinsic determinants, including *aph(3′)-IIb*, *fosA*, *catB7*, and the efflux genes, were conserved across most STs, whereas acquired-candidate genes, including carbapenemases (β-lactams), aminoglycoside-modifying enzymes (aminoglycosides), *sul1* (sulfonamides), tetracycline resistance genes, and macrolide resistance determinants, showed lineage-specific distributions. Members of the chromosomal *bla*_OXA_ and *bla*_PDC_ families (β-lactams) also showed ST-specific patterns, reflecting allelic diversity within these genes.

**Fig. 2.**
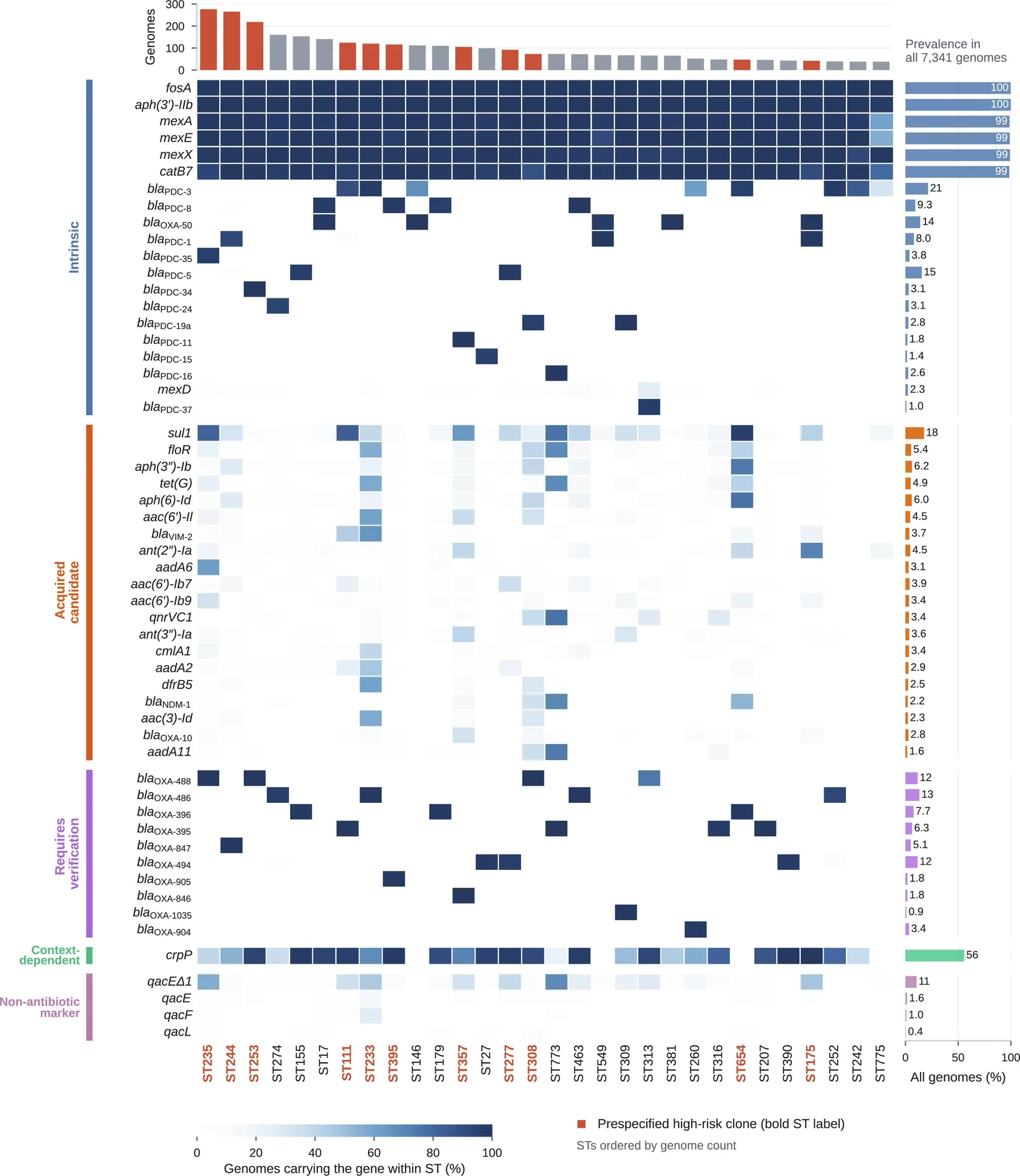
Prevalence of consensus ARGs among the 30 most frequent *P. aeruginosa* sequence types (STs). The heatmap displays the 20 most prevalent intrinsic determinants, the 20 most prevalent acquired-candidate ARGs, 10 determinants requiring further verification, and all context-dependent (*n* = 1) and non-antibiotic-marker (*n* = 4) genes. Genes are ranked within each category by the number of positive genomes among the 30 STs. Cell color indicates the percentage of genomes within each ST carrying the gene (0% to 100%); colored bands on the left indicate the ARG category (Table S2.2). The upper bar chart gives the number of genomes per ST (prespecified high-risk clones in red, also indicated by red ST labels), and the bars on the right give the prevalence of each gene among all 7,341 genomes. STs are ordered by genome count.

### Mobilome landscape and unadjusted associations with acquired-candidate ARG burden

IntegronFinder identified at least one integron-associated structure in 1,642 of the 7,341 genomes (22.4%) (Fig. 3A and Table 1). Complete integrons were detected in 1,056 genomes (14.4%), whereas CALIN structures and In0 elements were identified in 440 (6.0%) and 492 (6.7%) genomes, respectively. Overall, 1,539 genomes (21.0%) contained at least one *attC* site, and 1,472 (20.1%) carried at least one IntegronFinder-predicted integron-associated gene cassette (Table S4).

**Fig. 3.**
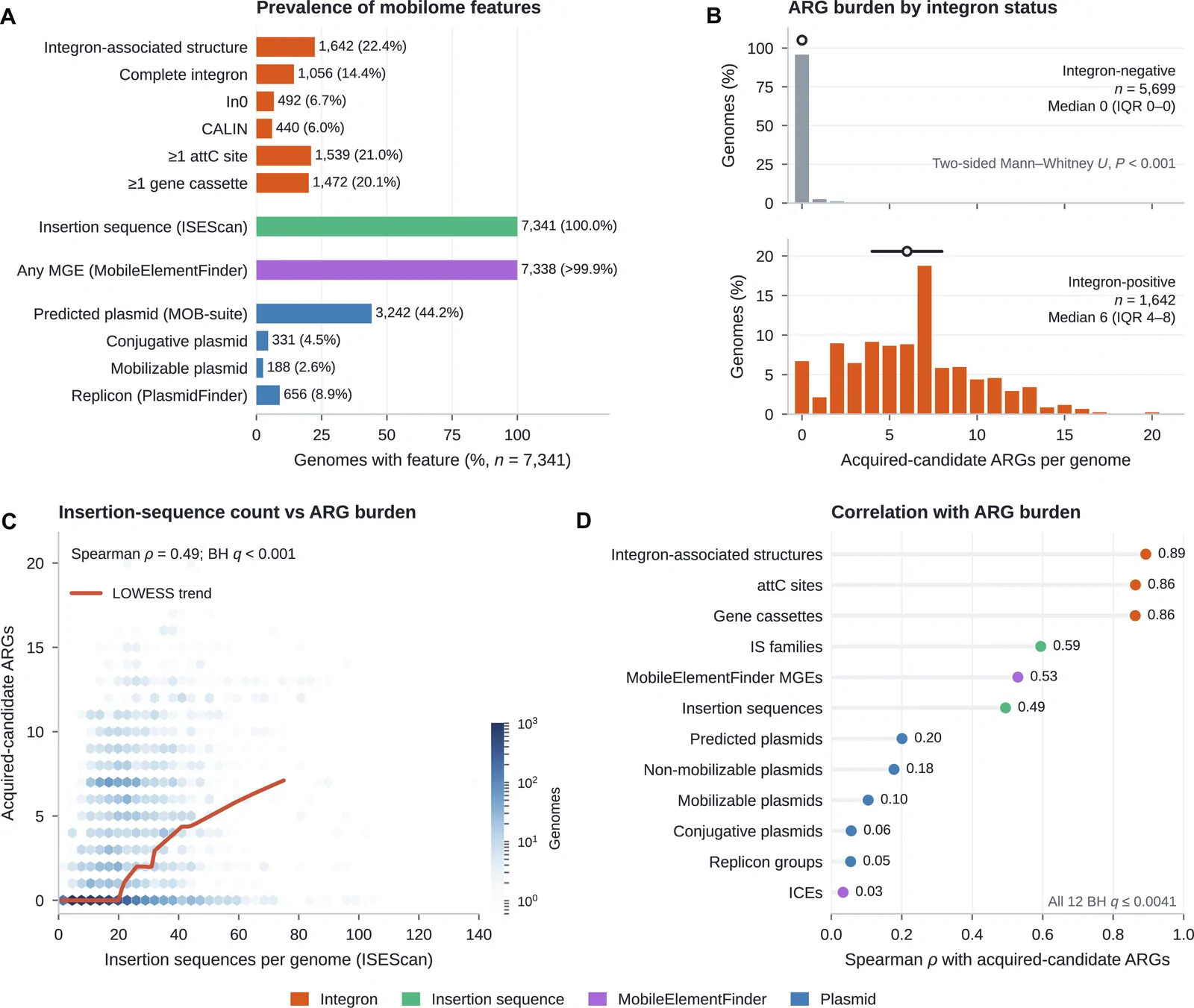
Mobilome features and acquired-candidate antimicrobial resistance gene (ARG) burden in 7,341 *P. aeruginosa* genomes. (A) Prevalence of the major mobilome features listed in Table 1, colored by element category. (B) Distribution of acquired-candidate ARG counts in integron-negative (*n* = 5,699) and integron-positive (*n* = 1,642) genomes; bars give the percentage of genomes in each group with each count, and the point and line show the median and interquartile range (2-sided Mann–Whitney *U* test, *P* < 0.001). (C) Insertion-sequence count versus acquired-candidate ARG count; hexagonal bins show the number of genomes (log scale) and the red line shows a robust LOWESS trend over the range containing 99.5% of genomes. (D) Spearman correlations of 12 prespecified mobilome features with acquired-candidate ARG count, colored by element category; all remained significant after Benjamini–Hochberg correction (*q* ≤ 0.0041; Table S5). The acquired-candidate outcome excludes intrinsic, context-dependent, unverified, and non-antibiotic-marker annotations.

**Table 1.** Prevalence of major genome-level mobilome features (*n* = 7,341). Complete integrons contain an integrase gene and associated *attC* sites; CALIN denotes a cluster of *attC* sites lacking an associated integrase gene; In0 denotes an integrase-containing structure without detected *attC* sites. Categories may co-occur within a genome. Plasmid predictions and mobility classifications are based on MOB-suite; replicon detection is based on PlasmidFinder. Absence from the hit-only PlasmidFinder results was coded as zero, assuming that all 7,341 genomes were screened. Percentages use 7,341 genomes as the denominator; 7,338/7,341 is shown as >99.9% to distinguish it from detection in all genomes.

Detection tool	Feature	Positive genomes	Positive (%)
IntegronFinder	Integron-associated structure	1,642	22.4
IntegronFinder	Complete integron	1,056	14.4
IntegronFinder	In0	492	6.7
IntegronFinder	CALIN	440	6.0
IntegronFinder	At least one *attC* site	1,539	21.0
IntegronFinder	At least one gene cassette	1,472	20.1
ISEScan	Insertion sequences detected	7,341	100.0
MobileElementFinder	Mobile genetic element detected	7,338	>99.9
MOB-suite	Predicted plasmid	3,242	44.2
MOB-suite	Conjugative plasmid	331	4.5
MOB-suite	Mobilizable plasmid	188	2.6
PlasmidFinder	Replicon detected	656	8.9

ISEScan detected ISs in all 7,341 genomes (100.0%). MobileElementFinder identified at least one reference-associated MGE in 7,338 genomes (>99.9%). MOB-suite reconstructed one or more predicted plasmids in 3,242 genomes (44.2%), including 331 genomes (4.5%) carrying at least one predicted conjugative plasmid and 188 (2.6%) carrying at least one predicted mobilizable plasmid. PlasmidFinder reported known plasmid replicons in 656 genomes (8.9%) (Fig. 3A, Table 1, and Table S5). Replicon detection was treated separately from MOB-suite plasmid reconstruction.

Integron-positive genomes carried a greater acquired-candidate ARG burden than integron-negative genomes (Fig. 3B). The median acquired-candidate count was 6 genes (IQR, 4 to 8) in integron-positive genomes (*n* = 1,642), compared with 0 (IQR, 0 to 0) in integron-negative genomes (*n* = 5,699; 2-sided Mann–Whitney *U* test, *P* < 0.001). Mean counts were 6.289 and 0.075, respectively. Total consensus counts, analyzed as a secondary description, had medians of 16 and 9 in these groups.

Consensus acquired-candidate ARG burden was positively correlated with multiple mobilome features (Fig. 3C and D). The strongest correlations were observed for integron-associated structure count (Spearman *ρ* = 0.89), *attC*-site count (*ρ* = 0.86), and gene-cassette count (*ρ* = 0.86). Positive correlations were also observed for IS-family diversity (*ρ* = 0.59), MobileElementFinder burden (*ρ* = 0.53), total IS count (*ρ* = 0.49), predicted plasmid count (*ρ* = 0.20), non-mobilizable plasmid count (*ρ* = 0.18), mobilizable plasmid count (*ρ* = 0.10), conjugative plasmid count (*ρ* = 0.06), replicon-group count (*ρ* = 0.05), and ICE count (*ρ* = 0.03). All 12 Spearman correlations remained statistically significant after Benjamini–Hochberg false-discovery-rate correction (*q* ≤ 0.00412). The strongest associations were observed for integron-associated structure count (*ρ* = 0.89), *attC*-site count (*ρ* = 0.86), and gene-cassette count (*ρ* = 0.86), all with *q* < 0.001. Individual raw *P* values and BH-adjusted *q* values are provided in Table S5. These unadjusted correlations were not interpreted as independent effects. IS-family counts represented unique non-unknown family labels per genome, and replicon-type counts represented unique replication groups.

### Adjusted associations between mobilome features and acquired-candidate ARG burden

After mutual adjustment for all mobilome features, country group, and assembly fragmentation, and accounting for clustering within STs (6,951 genomes in 1,169 ST clusters), the presence of an integron-associated structure remained the strongest correlate of acquired-candidate ARG burden, with an approximately 42-fold higher expected count (aRR 42.034, 95% CI 26.934 to 65.599; Table 2 and Fig. S1). Each 1-SD increase in log-transformed MobileElementFinder count was associated with a 35% higher expected count (aRR 1.349, 95% CI 1.188 to 1.531), and each additional MOB-suite-predicted plasmid was associated with a 13% higher count (aRR 1.129, 95% CI 1.069 to 1.192); PlasmidFinder replicon presence showed a similarly modest association (aRR 1.310, 95% CI 1.028 to 1.669). In contrast, IS count was not independently associated with acquired-candidate ARG burden once the other features were included (aRR 1.014, 95% CI 0.896 to 1.149), despite its positive unadjusted correlation (*ρ* = 0.49), indicating that its crude association largely overlapped with integron and other mobile-element features. Presence of a predicted conjugative plasmid (aRR 0.792, 95% CI 0.677 to 0.927) and ICE count (aRR 0.905, 95% CI 0.823 to 0.996) were associated with slightly lower expected counts, and assembly fragmentation was not associated with the outcome (aRR 0.987, 95% CI 0.920 to 1.060). Multicollinearity among the mobilome and assembly predictors was low (all variance inflation factors < 4), and the Pearson dispersion statistic (approximately 2.1) indicated moderate overdispersion, which is accommodated by the robust sandwich standard errors. These adjusted estimates describe differences in expected gene counts and do not establish physical linkage or transfer of ARGs.

**Table 2.** Associations between mobilome features and acquired-candidate ARG burden in a multivariable Poisson GEE model clustered by sequence type. The primary model included 6,951 assigned-ST genomes across 1,169 ST clusters, with an exchangeable working-correlation structure and robust sandwich standard errors. All 7 mobilome predictors were fitted simultaneously with standardized log(contig count + 1) and country group. Country groups were defined by a threshold of at least 50 assigned-ST genomes, with separate Other and Unknown groups. IS, MobileElementFinder, and contig counts were log(count + 1)-transformed and standardized across all 7,341 genomes; their aRRs are per 1-SD increase. Other counts are per additional element, and binary predictors compare presence with absence. *P* values are nominal 2-sided Wald *P* values. Confidence limits are shown to 3 decimal places. The assembly-fragmentation covariate is included in the table; complete country coefficients and sensitivity results are provided in the accompanying supplementary statistical files.

Predictor	Adjusted rate ratio	95% CI	*P* value
Integron-associated structure present	42.034	26.934–65.599	<0.001
log(IS count + 1), per SD	1.014	0.896–1.149	0.822
log(MEF count + 1), per SD	1.349	1.188–1.531	<0.001
Predicted plasmid count	1.129	1.069–1.192	<0.001
Conjugative plasmid present	0.792	0.677–0.927	0.004
ICE count	0.905	0.823–0.996	0.041
PlasmidFinder replicon present	1.310	1.028–1.669	0.029
log(contig count + 1), per SD	0.987	0.920–1.060	0.727

### Coordinate-based ARG–MGE colocalization

Coordinate-based analysis identified 10,752 acquired-candidate ARG genome–gene records with available positions across the 7,341-genome dataset. Of these, 4,303 (40.02%) overlapped inferred integron-associated spans, including 2,734 (25.43%) overlapping structures classified as complete integrons and 1,569 (14.59%) overlapping CALINs. Additionally, 3,280 (30.51%) and 6,433 (59.83%) were located within 1 and 5 kb of annotated ISs, respectively, while 11 (0.10%) overlapped predicted ICE intervals.

A sensitivity analysis restricted to 1,367 complete genomes included 3,297 coordinate-bearing acquired-candidate ARG records. The proportion overlapping complete-integron spans was similar to that in the full dataset (24.20%), whereas CALIN overlap was lower (4.43%) and IS proximity within 5 kb was higher (85.62%). Predicted ICE overlap remained infrequent (0.15%). These results indicate computational colocalization with annotated mobile-element contexts; they do not establish gene-cassette membership, plasmid carriage, or transfer events (Table S6).

### Geographic and temporal distribution of carbapenemases

Carbapenemase genes were detected in 997 genomes (13.6%), most often VIM-family (*n* = 368), IMP-family (*n* = 218), KPC-family (*n* = 195), and NDM-family (*n* = 160) genes, followed by GES carbapenemase variants (*n* = 82), SPM-family (*n* = 24), and other families (*n* = 18). Family composition differed between countries (Fig. 4A and C): VIM-family genes predominated in Taiwan and in several European countries; NDM-family genes predominated in Thailand, Singapore, and India; KPC-family genes predominated in China (together with IMP- and VIM-family genes) and Brazil; and 22 of the 24 SPM-positive genomes originated from Brazil. Among the 35 countries contributing at least 20 genomes, prespecified high-risk clones ranked among the 5 most frequent STs in 31 (Fig. 4B). Among 5,881 genomes collected between 2000 and 2024, the proportion carrying any carbapenemase increased from a fitted 4.1% in 2000 to 24.7% in 2024 (odds ratio per year 1.09, 95% CI 1.07 to 1.11; Fig. 5B). Compared with 2000 to 2012, genomes collected in 2018 to 2024 more often carried VIM-family (7.5% *vs.* 3.7%), IMP-family (4.6% *vs.* 1.6%), KPC-family (4.4% *vs.* 0.3%), and NDM-family genes (3.7% *vs.* 0.0%), whereas SPM-family genes became rarer (0.1% *vs.* 0.8%) (Fig. 5C). Because submission intensity varied strongly across countries and years, these values describe the public-genome collection rather than population prevalence.

**Fig. 4.**
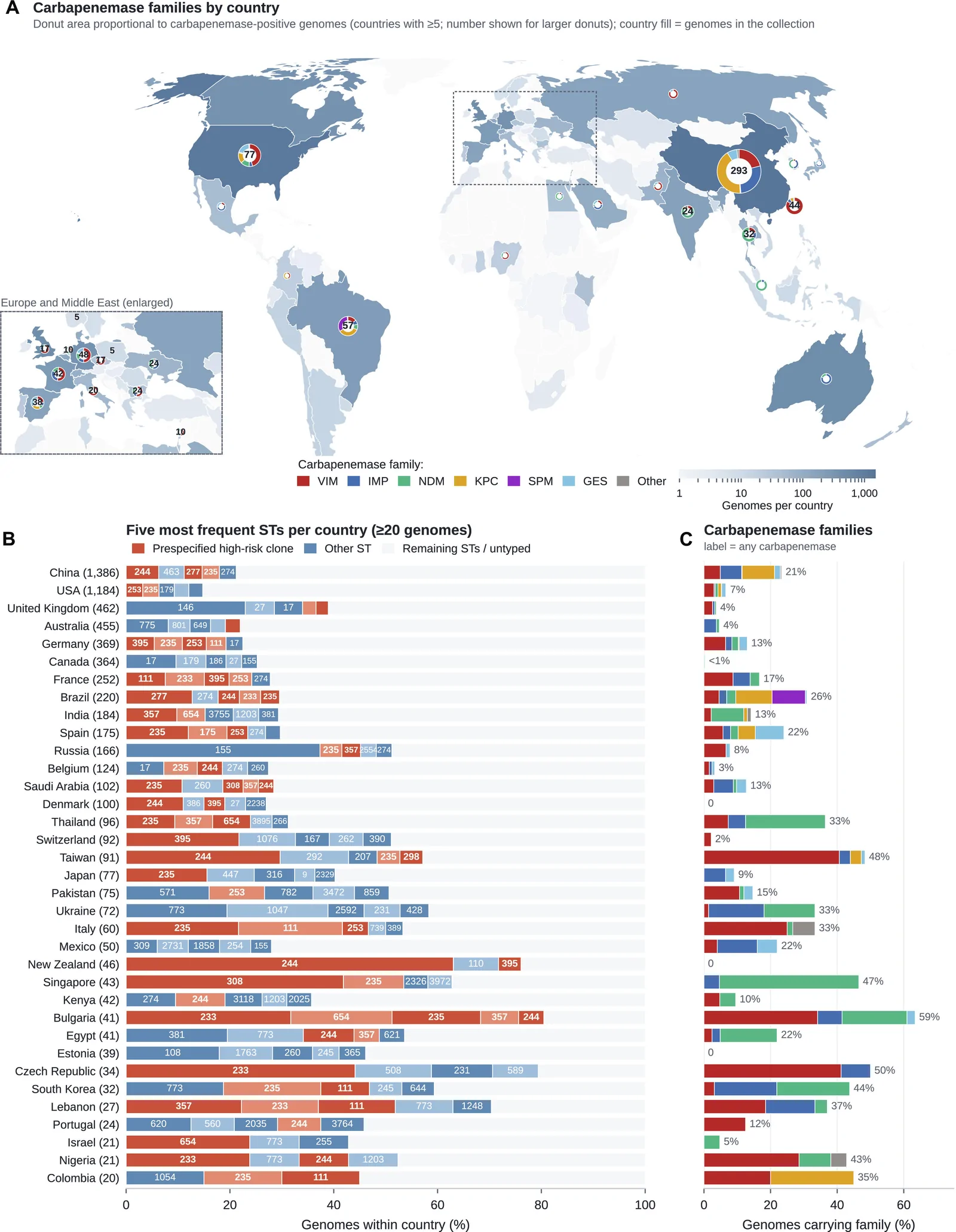
Geographic distribution of carbapenemase gene families and sequence types among *P. aeruginosa* genomes. (A) World map (Equal Earth projection); country fill indicates the number of genomes in the collection, and donuts show the carbapenemase-family composition of carbapenemase-positive genomes for the 29 countries with at least 5 such genomes. Donut area is proportional to the number of carbapenemase-positive genomes (given for larger donuts); Europe and the Middle East are enlarged in the inset. (B) The 5 most frequent STs within each of the 35 countries contributing at least 20 genomes; segment width gives the percentage of the country’s genomes, prespecified high-risk clones are shown in red, and STs represented by a single genome are not ranked. (C) Percentage of each country’s genomes carrying each carbapenemase family; labels give the percentage carrying any carbapenemase, and a genome carrying 2 families contributes to both segments. Families are defined in the “Consensus resistome analysis” section. Genomes without country information (*n* = 485) and 9 marine samples are not shown. Values describe the public-genome collection and are not estimates of country-level prevalence; country-level counts are provided in the accompanying source-data files.

**Fig. 5.**
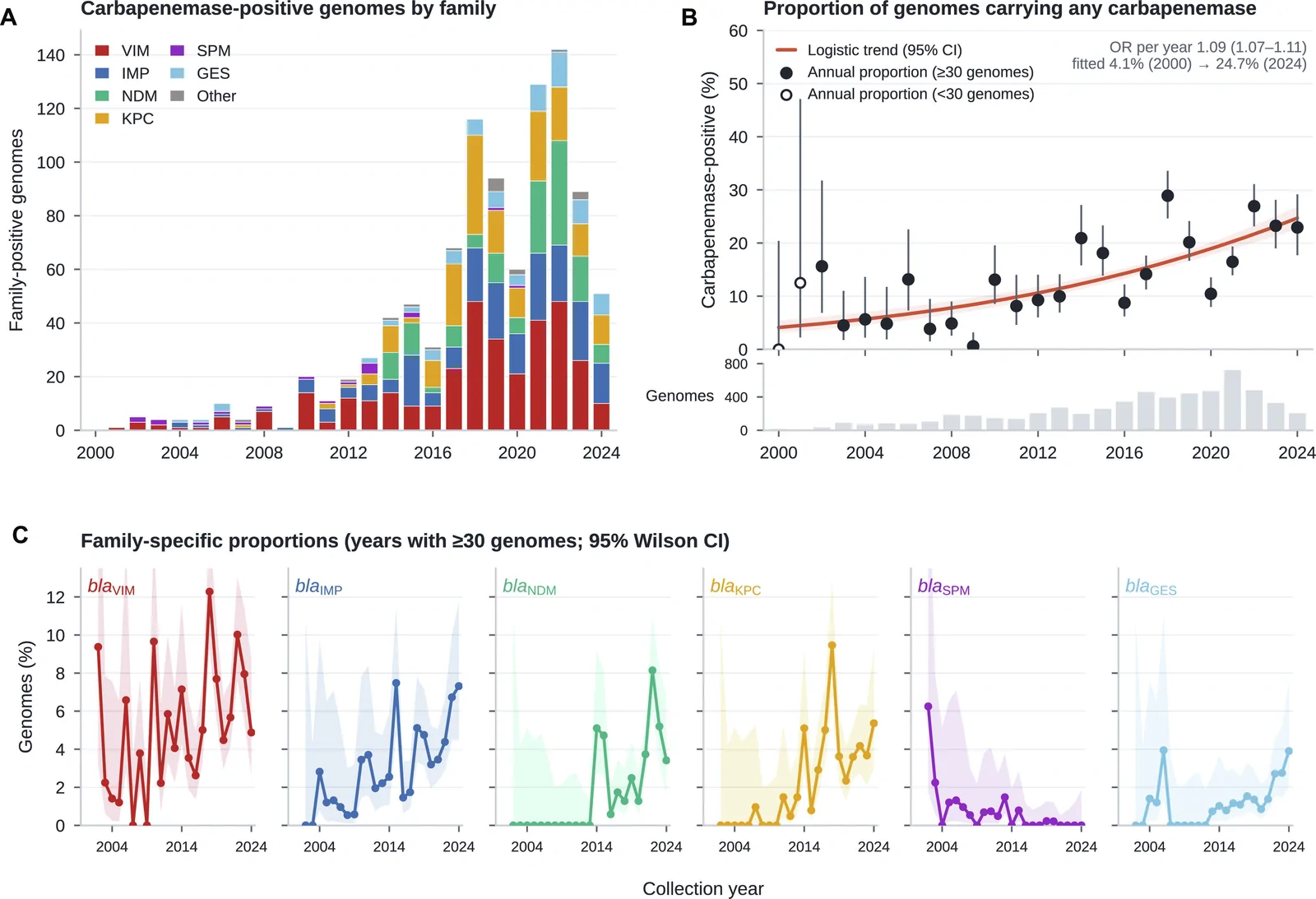
Carbapenemase genes among *P. aeruginosa* genomes by collection year, 2000 to 2024 (*n* = 5,881 genomes). (A) Number of genomes carrying each carbapenemase family per year; genomes carrying 2 families are counted in both. (B) Annual proportion of genomes carrying any carbapenemase with 95% Wilson CIs (open symbols: years with fewer than 30 genomes) and a genome-level logistic-regression trend with its 95% confidence band (odds ratio per year 1.09, 95% CI 1.07 to 1.11); the lower bar chart gives the number of genomes per collection year. (C) Annual proportion of genomes carrying each major carbapenemase family, shown for years with at least 30 genomes, with 95% Wilson CIs. Proportions describe the composition of the available public-genome collection and are not unbiased estimates of prevalence.

### Conserved and lineage-variable virulence profiles

A total of 141 curated virulence-associated genes were identified. Of these, 97 (68.8%) were classified as core, 22 (15.6%) as near-core, 12 (8.5%) as accessory, and 10 (7.1%) as rare (Fig. 6A). Each rare gene (*iucABC*/*iutA*, *cdtABC*, *papG*/*papX*, and *CT847*) was detected in a single genome (4 genomes in total; Table S7) and was not interpreted further. Selected virulence markers from the curated matrix showed lineage-specific distributions across the 30 most prevalent STs (*n* = 2,968 genomes; Fig. 6B). The type III secretion effector genes *exoS* and *exoU* displayed a largely reciprocal distribution. Specifically, ST235, ST253, ST357, ST308, ST773, ST309, ST313, ST316, and ST207 were predominantly *exoU*-positive, whereas ST244, ST274, ST155, ST17, ST111, ST233, ST395, ST146, ST179, ST27, ST277, ST549, ST381, ST260, ST654, ST390, ST175, ST252, ST242, and ST775 were predominantly *exoS*-positive. ST463 showed a distinctive profile in which both *exoS* and *exoU* were co-detected. Other effector and toxin genes were broadly conserved across dominant STs (*exoT* ≥97.5%, *lasB* ≥99.3%, and *pvdA* ≥94.7% in every ST), with notable exceptions in *exoY*, which was absent from ST309 and reduced in ST313 (86%), and *toxA*, which was present in only 48% of ST277 genomes (Fig. 6B). A dedicated BLASTP screening for exolysin A (*exlA*) identified hits in 44 genomes (0.6%; 94.5 to 95.0% amino acid identity to PA7 ExlA, 100% query coverage), which were distributed across 23 STs, lacked all T3SS effectors (*exoS*, *exoU*, *exoT*, and *exoY*), and did not belong to the 30 most prevalent STs (Table S7).

**Fig. 6.**
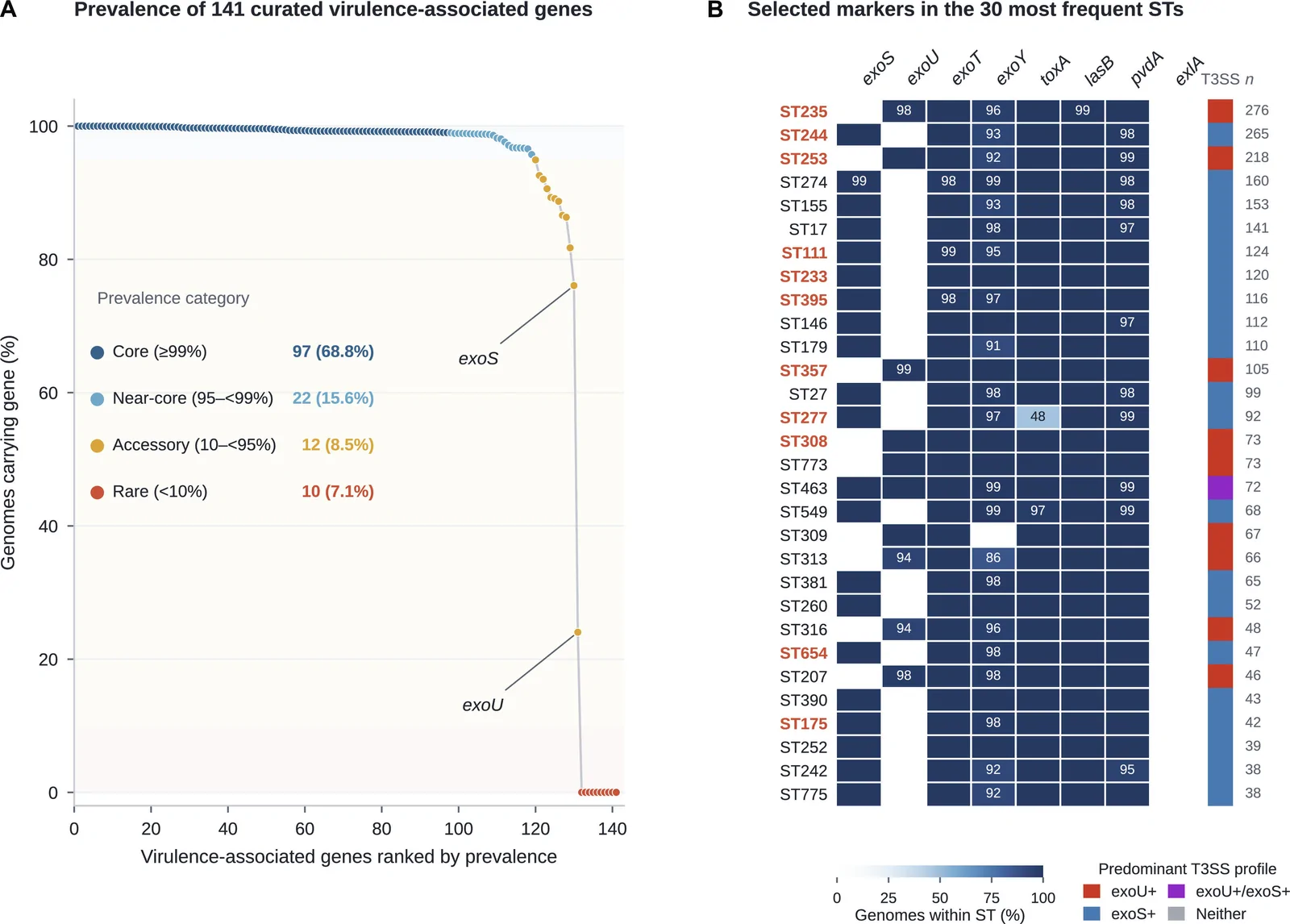
Virulence-associated gene profiles across *P. aeruginosa* sequence types. (A) Prevalence of the 141 curated virulence-associated genes ranked from most to least prevalent and colored by prevalence category: core (≥99%), near-core (95% to <99%), accessory (10% to <95%), and rare (<10%). (B) Prevalence of selected virulence-associated genes among the 30 most frequent STs (*n* = 2,968 genomes). Cell color indicates the percentage of genomes within each ST carrying the gene, and values are printed for cells between 1% and 99%; *exlA* (BLASTP screen; see the “Virulence-associated gene analysis” section) was not detected in any of these STs. The strip on the right gives the predominant T3SS effector profile of each ST (*exoU* and/or *exoS* detected in ≥50% of genomes) and the number of genomes; prespecified high-risk clones are labeled in red.

The combined distribution of ARG and virulence-associated gene burden across major STs is summarized in Fig. S2; across the 66 STs with at least 20 genomes, mean acquired-candidate ARG burden was not correlated with mean virulence-associated gene content (Spearman *ρ* = 0.13, *P* = 0.28).

### Phylogenomic distribution of resistance and virulence traits

Core-genome phylogenetic analysis of 7,341 *P. aeruginosa* genomes resolved multiple lineages, including clusters containing the prespecified high-risk STs ST235, ST111, ST233, ST244, ST357, ST308, ST175, ST277, ST654, ST298, ST253, and ST395 (Fig. 7A). Consensus ARG counts varied across the phylogeny. Predicted carbapenemase gene families, including VIM, IMP, NDM, and KPC, were detected in different parts of the tree rather than being uniformly distributed across lineages.

**Fig. 7.**
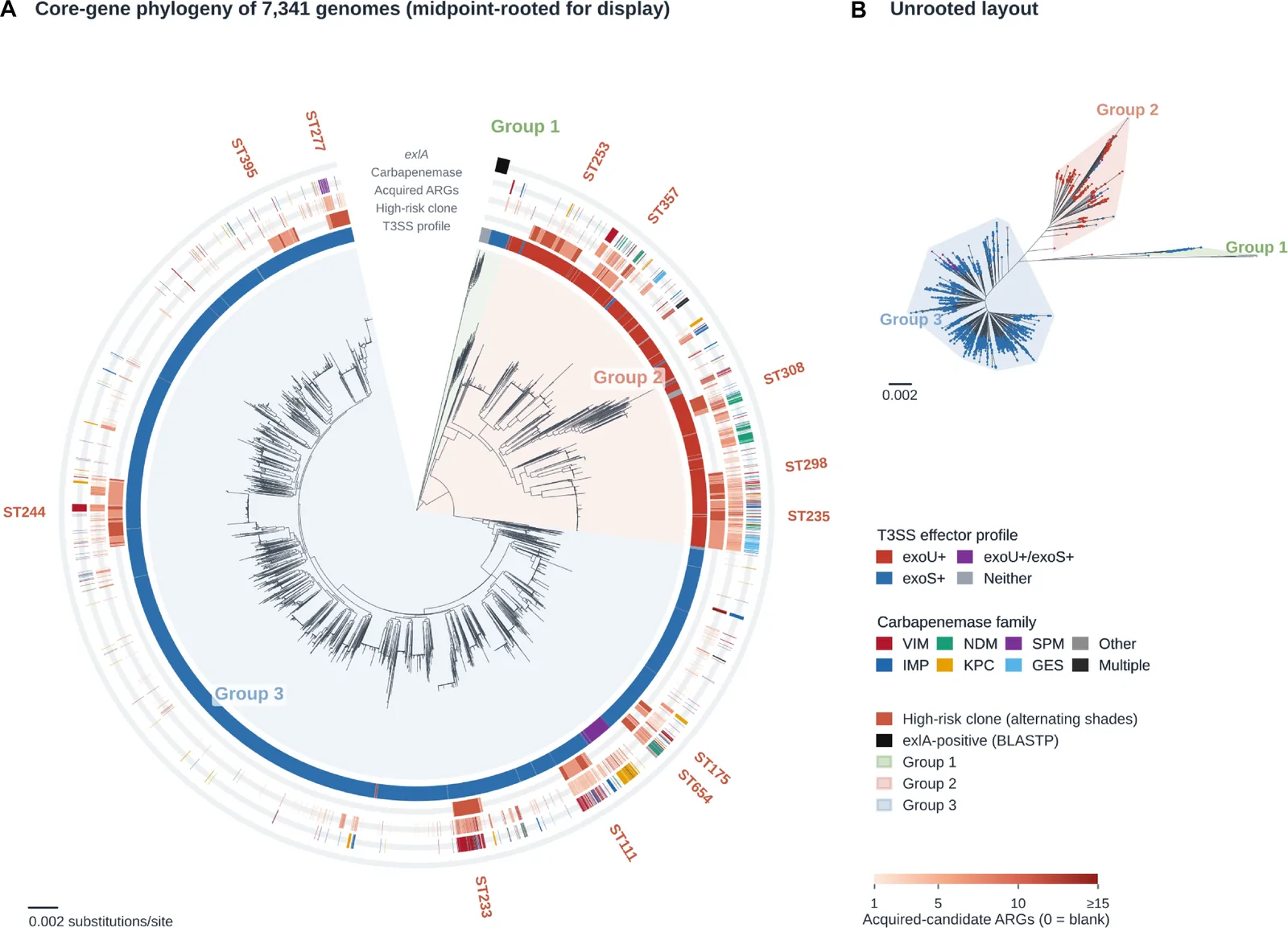
Core-gene maximum-likelihood phylogeny of 7,341 *P. aeruginosa* genomes. (A) Circular layout of the IQ-TREE2 tree inferred from the PIRATE core-gene alignment (see the “Phylogenomic analysis” section), midpoint-rooted for display only. Background shading marks Groups 1 to 3, defined by the 2 deepest splits of the midpoint-rooted tree (one genome branching outside Groups 2 and 3 was left unassigned). Rings, from inside to outside: T3SS effector profile (*exoU*+, *exoS*+, *exoU*+/*exoS*+, or neither); prespecified high-risk clones (alternating shades separate adjacent clones, which are labeled outside the rings); acquired-candidate ARG count (blank, none); carbapenemase family (see the “Consensus resistome analysis” section; Multiple, more than one family); and *exlA*-positive genomes (BLASTP; see the “Virulence-associated gene analysis” section). (B) Unrooted equal-angle layout of the same tree, with tips colored by T3SS effector profile and Groups 1 to 3 shaded. Scale bars, substitutions per site. Group membership of each genome is provided in the source-data files.

T3SS effector profiles also differed among phylogenetic groupings. *exoU*-positive and *exoS*-positive genomes were concentrated in separate regions of the circular phylogeny (Fig. 7A), with a corresponding separation visible in the unrooted tree (Fig. 7B). *exlA* was detected in a subset of genomes lacking both *exoU* and *exoS* (Fig. 7A). The midpoint-rooted tree comprised 3 groups (Fig. 7): Group 1 (*n* = 127), a divergent lineage that contained all 44 *exlA*-positive genomes, which lacked *exoS*, *exoU*, *exoT*, and *exoY*, while the remaining 83 genomes were *exoS*-positive; Group 2 (*n* = 1,726), in which 94.4% of genomes were *exoU*-positive only; and Group 3 (*n* = 5,487), in which 97.3% were *exoS*-positive only. Carbapenemase genes were detected in all 3 phylogenetic groups (Group 1, 11 genomes; Group 2, 410; Group 3, 576).

## Discussion

This study integrated population structure, a 3-tool consensus resistome, complementary mobilome analyses, virulence profiling, and lineage-aware statistical modeling across 7,341 quality-filtered *P. aeruginosa* genomes. Four major findings emerged. First, the public-genome collection was highly diverse but enriched for a limited number of widely disseminated STs. Second, integron-associated features showed the strongest associations with acquired-candidate ARG burden, whereas IS-related metrics exhibited moderate unadjusted correlations. Total IS count was not independently associated with ARG burden after multivariable adjustment. Third, plasmid-associated measures exhibited comparatively weaker associations with ARG burden. Finally, virulence-associated gene content was largely conserved, whereas the accessory effectors *exoU* and *exoS* displayed strong lineage-specific distributions.

An additional strength of this study is the use of a consensus resistome framework integrating 3 complementary AMR databases. Previous comparative genomic studies have often relied on a single annotation resource, despite known inconsistencies in gene nomenclature and database content. Requiring agreement between tools aimed to limit resource-specific calls, but could exclude genuine determinants absent from or named differently across databases. Agreement between these resources should not be treated as independent validation. Recent pangenome-based work further supports this limitation. Do *et al.* [34] showed that unitig-centered genomic features in *P. aeruginosa* can capture resistance-associated signals beyond conventionally annotated ARGs, indicating that gene-based resistome screening may not encompass the full genomic basis of AMR. Emerging pan-genomic feature-selection approaches, such as PanARGMiner, may further facilitate the identification of both known and previously unrecognized resistance-associated biomarkers beyond predefined ARG catalogs [35].

The MLST analysis demonstrated substantial global diversity within the *P. aeruginosa* population, with more than 1,000 STs identified across the dataset. The predominance of ST235, ST244, ST253, ST274, ST155, ST111, ST233, and related lineages is consistent with the established role of epidemic and high-risk clones in *P. aeruginosa* dissemination [11–13]. However, the large number of distinct STs and the distribution of carbapenemase genes across multiple phylogenetic lineages indicate that clinically important AMR is not confined to a limited number of successful clones. These findings highlight the importance of genomic surveillance strategies that monitor both established high-risk clones and emerging local lineages. Recent population-genomic studies provide additional context for this lineage structure. Abram *et al.* [36] showed that the *P. aeruginosa* pangenome comprises a substantial accessory component and identified 3 major phylogroups together with multiple genomic substructures, emphasizing the contribution of accessory-gene content to population differentiation. Botelho *et al.* [37] further demonstrated phylogroup-specific clustering of AMR determinants within regions of genome plasticity, indicating that resistance-associated accessory elements are structured by genomic background rather than being randomly distributed across the species. More recently, Weimann *et al.* [38] showed that globally successful epidemic clones have emerged through a combination of horizontal gene acquisition, clonal expansion, and host-specific adaptation. Together, these studies support interpretation of the present findings within a population-genomic framework in which clonal background and accessory-genome dynamics jointly shape resistance-gene distribution.

The high prevalence of *aph(3′)-IIb*, *fosA*, *catB7*, and the *mex* efflux system components reflects their conserved, soft-core distribution in *P. aeruginosa* [7,39]. However, the detection of individual *mex* genes does not necessarily indicate phenotypic resistance, because efflux-mediated resistance depends on the integrity, expression, and regulation of the complete efflux pump system [7,40]*.* The ST-structured allelic diversity of the chromosomal *bla*_OXA_ and *bla*_PDC_ β-lactamase families is consistent with their species-associated, chromosomally encoded nature [41–43]. The strong association between specific PDC variants and particular STs is consistent with previous genomic studies showing that certain PDC alleles are closely linked to defined phylogenetic lineages [43,44]. Because of this stable association, lineage-specific β-lactamase profiles have been proposed as useful markers for differentiating major *P. aeruginosa* clones. In contrast, carbapenemase genes (e.g., *bla*_VIM-2_, *bla*_NDM-1_, and *bla*_KPC-2_), aminoglycoside-modifying enzymes, *sul1*, and tetracycline and macrolide resistance genes were detected at lower frequencies and displayed a more heterogeneous distribution across STs [45]. Unlike intrinsic β-lactamases, these enzymes are not consistently associated with certain lineages. Previous studies have reported that carbapenemase genes in *P. aeruginosa* are frequently carried on integrons and other MGEs, facilitating their occurrence across diverse genetic backgrounds. This likely explains why their distribution varies across STs rather than following stable lineage-associated patterns.

A central finding of this work is that acquired-candidate ARG burden is far more strongly correlated with integron and IS metrics than with plasmid-based measures in unadjusted analyses. In the acquired-candidate analysis, integron-positive genomes carried a median of 6 genes, compared with 0 in integron-negative genomes; the strongest unadjusted correlations were with integron-associated structure count (Spearman *ρ* = 0.89), *attC-*site count (*ρ* = 0.86), and gene-cassette count (*ρ* = 0.86). The positive unadjusted correlations with IS abundance and IS-family diversity are consistent with the established role of IS elements in mobilizing and amplifying resistance genes and in remodeling the accessory genome [46]. However, total IS count was not independently associated with acquired-candidate ARG burden after multivariable adjustment (aRR 1.014, 95% CI 0.896 to 1.149). Integrons capture and express gene cassettes, whereas IS elements can create composite structures, alter gene expression, and facilitate genomic rearrangements, providing biological context for the observed unadjusted associations. Plasmid-related unadjusted associations were weaker than integron and IS associations. In the adjusted model, each additional MOB-suite plasmid was associated with only a modest increase in expected ARG count, and known replicon presence showed a similarly modest adjusted association. The comparatively weaker associations observed for plasmid-based metrics should not be interpreted as indicating that plasmids are unimportant in *P. aeruginosa*. Recent work by Wang *et al.* showed that *P. aeruginosa* megaplasmids have distinct structural and phylogenetic characteristics and can carry clinically relevant resistance determinants [47]. Thus, the weaker plasmid–ARG associations observed here may reflect limitations of simple plasmid count or presence metrics rather than a minor role of plasmids in resistance dissemination.

Carbapenemase families displayed marked geographic and lineage structure. VIM-family genes were prominent across several European settings and Taiwan; NDM-family genes were frequent in genomes from Thailand, Singapore, India, and other Asian settings; KPC-family genes were concentrated in China and Brazil; and *bla*_SPM-1_ remained strongly associated with Brazilian genomes. These patterns support a model in which international clone dissemination is superimposed on regional acquisition and expansion [48–50]. The apparent rise in carbapenemase-positive genomes, from a fitted 4.1% in 2000 to 24.7% in 2024, is clinically concerning but must be interpreted cautiously, because RefSeq sampling is shaped by sequencing capacity, outbreak-driven submission, country representation, and clinical ascertainment. These temporal patterns should therefore be regarded as describing the available genome collection rather than as unbiased estimates of global prevalence; nonetheless, the increasing representation of *bla*_KPC_, *bla*_NDM_, and *bla*_IMP_ in recent years highlights the importance of monitoring carbapenemase diversity in addition to total carbapenem resistance.

The virulome exhibited a distinct pattern from the resistome. To improve the specificity of the VFDB-based analysis, we excluded 108 genes, comprising genes involved in motility, adhesion, and general cellular functions, including members of the *alg*, *chp*, *fim*, *fle*, *flg*, *fli*, *flh*, *mot*, *muc*, and *pil* operons, and the pyoverdine-locus genes *pvdD*, *pvdI*, and *pvdJ*, whose apparent presence or absence reflects allelic divergence rather than gene loss. The resulting curated set comprised 141 virulence-associated genes, most of which were classified as core or near-core. Unlike the resistome, the total number of curated virulence-associated genes showed little correlation with ARG burden. Differences among major lineages were primarily associated with T3SS effector profiles, particularly the largely reciprocal distribution of *exoU* and *exoS*, rather than variation in overall curated gene content. However, gene presence–absence profiles cannot capture allelic diversity, regulatory variation, or functional differences, and a larger repertoire of virulence-associated genes does not necessarily indicate greater pathogenicity. ST463 was notable for the co-detection of *exoU* and *exoS* and its elevated carbapenemase burden, although these genomic findings require phenotypic and clinical evidence to establish their implications for virulence [51]. Independent screening for exolysin A (*exlA*), which was not represented in the curated VFDB-based analysis, identified 44 genomes (0.6%) with high sequence similarity to the PA7 ExlA reference (94.5% to 95.0% amino acid identity and 100% query coverage). These genomes were distributed across 23 STs and were absent from the dominant lineages, suggesting that *exlA* is associated with relatively uncommon phylogenetic backgrounds in this collection. All 44 *exlA*-positive genomes lacked the T3SS effector genes *exoS*, *exoU*, *exoT*, and *exoY*, consistent with the previously described association between exolysin-producing strains and T3SS-negative genomic backgrounds [52,53]. Geographically, most *exlA*-positive genomes originated from Europe (34/44), including 13 from the United Kingdom. Animal-associated isolates accounted for 22.7% (10/44) of *exlA*-positive genomes, compared with 3.1% of the overall collection.

Global structure genome mapping with resistance and virulence determinants onto the core-genome phylogeny revealed contrasting evolutionary patterns. Consistent with the population structure shown in Fig. 7, the phylogeny resolved 3 groups: 2 major groups—a predominantly *exoS*-positive group (Group 3) and a predominantly *exoU*-positive group (Group 2)—and a smaller divergent lineage (Group 1) that contained all *exlA*-positive genomes. The distribution of the T3SS effectors *exoS* and *exoU* closely followed this structure, consistent with their long-term maintenance through vertical inheritance. This observation is consistent with previous studies showing that *exoS* and *exoU* are largely mutually exclusive and strongly associated with specific clonal lineages [54,55].

In contrast, carbapenemase genes and elevated ARG burdens were concentrated within several globally disseminated high-risk clones but were also distributed across multiple phylogenetic lineages, a distribution consistent with recurrent horizontal acquisition of resistance determinants in diverse genomic backgrounds. This pattern is consistent with the major role of MGEs in the dissemination of AMR in *P. aeruginosa* [55,56].

Together, these findings are consistent with lineage-associated *exoS*/*exoU* profiles and mobile-element-associated ARG accumulation across diverse *P. aeruginosa* lineages; they do not establish a transfer history.

This public RefSeq collection is strongly enriched for human-associated isolates and is shaped by uneven sequencing effort across countries, lineages, and years. We did not query ENA independently for assemblies, and duplicate-isolate curation does not eliminate outbreak or BioProject clustering. Draft assemblies can fragment integrons, ISs, and plasmids, so differences among mobilome metrics may partly reflect detectability. Gene detection omits many mutation-mediated resistance mechanisms and was not validated against antimicrobial susceptibility data; the acquired-candidate classification also depends on database coverage and gene-name normalization. Coordinate overlap and proximity are computational associations and do not prove cassette membership, plasmid carriage, transfer, or evolutionary direction. ST-clustered models only partly account for relatedness, and geographic or temporal frequencies in this dataset should not be interpreted as population prevalence. These limitations constrain inference about clinical resistance and mechanisms of horizontal gene transfer.

## Conclusions

This study shows that acquired-candidate ARG burden in the sampled *P. aeruginosa* genomes varies with clonal background and mobile-element measures, with integron-associated features showing the strongest associations with ARG burden across 7,341 genomes. IS-related metrics showed moderate unadjusted correlations, although total IS count was not significantly associated with ARG burden after multivariable adjustment. In contrast, plasmid-associated measures showed comparatively weaker unadjusted correlations, whereas virulence-associated gene content remained largely conserved, with lineage-specific differentiation primarily reflected in the distribution of *exoU* and *exoS*. Together, these observations are consistent with mobile-element-associated ARG accumulation across multiple lineages. The present analyses do not establish the direction or frequency of transfer events. This work provides a foundation for lineage-aware, mobilome-informed genomic surveillance and highlights complete genome-based ARG–MGE mapping as an important direction for future research.

## Ethical Approval

This study analyzed publicly available bacterial genome sequences and their associated metadata deposited in the NCBI RefSeq database. The project was reviewed by the Human Research Ethics Committee of the Faculty of Medicine, Prince of Songkla University (Ethics Approval No. REC.69-482-38-2).

## Data Availability

Assembly accessions and curated metadata are provided in Table S1. Per-tool ARG calls and normalized identifiers are provided in Table S2, with gene classifications provided in Table S2.2; consensus ARG results are provided in Table S3. Integron annotations and mobilome data are provided in Tables S4 and S5, respectively. Coordinate-based ARG–MGE comparisons are provided in Table S6, and the curated virulence results are provided in Table S7.
