# Supplementary material for: Global Population Genomics Links Mobilome Architecture to Antimicrobial Resistance Gene Burden in Pseudomonas aeruginosa
Source: Comput Struct Biotechnol J. 2026 Nov 5;35(1):0249. doi: 10.34133/csbj.0249 (PMC13735188; doi:10.34133/csbj.0249)

Mobilome features and acquired-candidate ARG burden: multivariable Poisson GEE

6,951 assigned-ST genomes in 1,169 ST clusters; exchangeable working correlation, robust SEs; all predictors fitted jointly, adjusted for country group and log(contig count + 1). Open symbols: 95% CI includes 1.

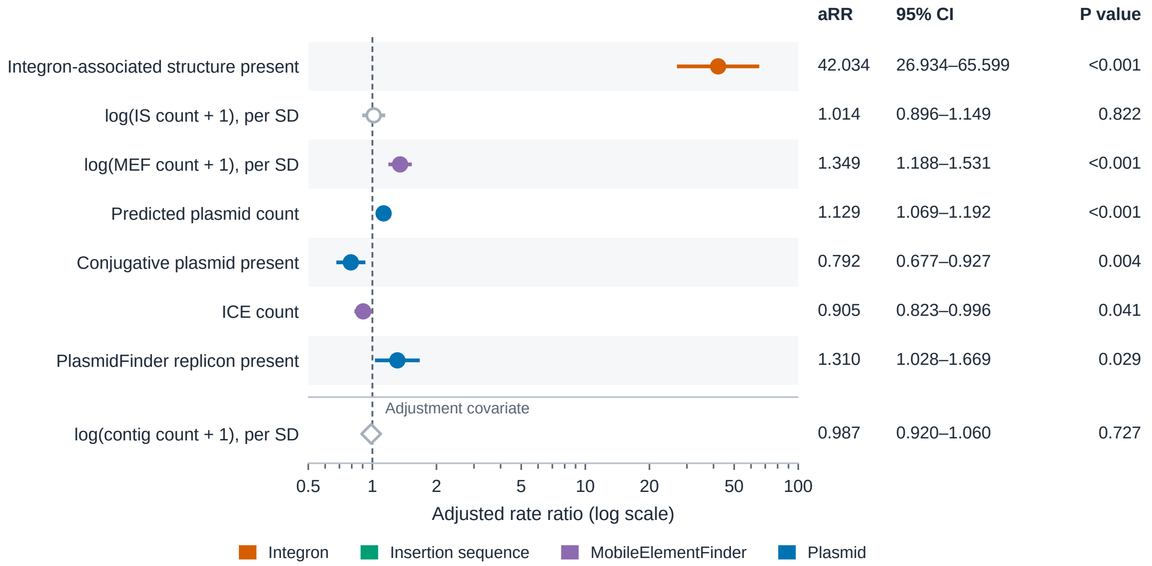

Supplement: Supplementary file 1 [file csbj.0249.f1.zip › Figure_S1.pdf]
