# Supplementary material for: Global Population Genomics Links Mobilome Architecture to Antimicrobial Resistance Gene Burden in Pseudomonas aeruginosa
Source: Comput Struct Biotechnol J. 2026 Nov 5;35(1):0249. doi: 10.34133/csbj.0249 (PMC13735188; doi:10.34133/csbj.0249)
Supplement: Supplementary file 1 [file csbj.0249.f1.zip › Figure_S2.pdf]

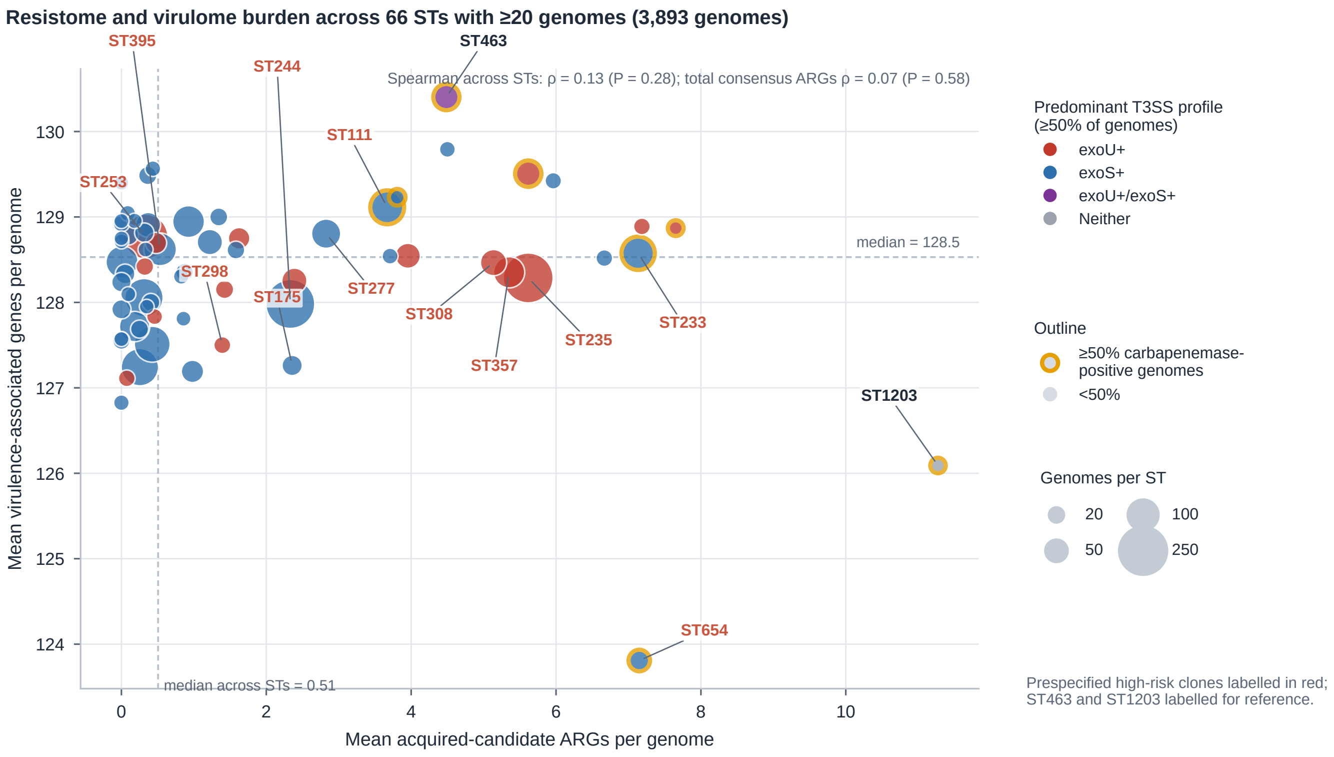
Resistome and virulome burden across 66 STs with ≥20 genomes (3,893 genomes)
Spearman across STs: ρ = 0.13 (P = 0.28); total consensus ARGs ρ = 0.07 (P = 0.58)
ST395
ST244
ST463
ST111
ST253
ST298
ST175
ST277
ST308
ST357
ST235
ST233
ST1203
ST654
median = 128.5
median across STs = 0.51
Mean virulence-associated genes per genome
Mean acquired-candidate ARGs per genome
130
129
128
127
126
125
124
0
2
4
6
8
10
Predominant T3SS profile (≥50% of genomes)
exoU+
exoS+
exoU+/exoS+
Neither
Outline
≥50% carbapenemase-positive genomes
<50%
Genomes per ST
20
100
50
250
Prespecified high-risk clones labelled in red; ST463 and ST1203 labelled for reference.
