# Supplementary material for: Global Population Genomics Links Mobilome Architecture to Antimicrobial Resistance Gene Burden in Pseudomonas aeruginosa
Source: Comput Struct Biotechnol J. 2026 Nov 5;35(1):0249. doi: 10.34133/csbj.0249 (PMC13735188; doi:10.34133/csbj.0249)
Supplement: Supplementary file 1 [file csbj.0249.f1.zip › Supplementary materials.docx]

# Supplementary materials

**Supplementary Figure S1. Adjusted associations of mobilome features with acquired-candidate ARG burden.** Points and horizontal lines show adjusted rate ratios (aRRs) and 95% confidence intervals from the primary Poisson GEE model (Table 2) of 6,951 genomes in 1,169 ST clusters, with an exchangeable working-correlation structure, robust standard errors and adjustment for country group; the assembly-fragmentation covariate, log(contig count + 1), is shown separately (diamond). Colours indicate mobilome category, and open grey symbols denote 95% CIs that include 1. The dashed line indicates a ratio of 1 and the x-axis is logarithmic. aRRs compare expected gene counts, not probabilities of resistance or horizontal-transfer events.

**Supplementary Figure S2. Integrated resistome and virulome profiles across major *P. aeruginosa* lineages.** Each bubble represents one of the 66 sequence types (STs) with at least 20 genomes (3,893 genomes). The horizontal axis gives the mean number of acquired-candidate ARGs per genome and the vertical axis the mean number of curated virulence-associated genes per genome; dashed lines mark the medians across STs, and bubble area is proportional to the number of genomes. Bubble colour indicates the predominant T3SS profile: *exoU*+ or *exoS*+ when the gene was detected in ≥50% of the ST’s genomes, *exoU*+/*exoS*+ when both were, and neither otherwise. Orange outlines mark STs in which ≥50% of genomes carried a carbapenemase gene (Section 2.3). Mean ARG and virulence-gene burdens were not correlated across STs (Spearman ρ = 0.13, *P* = 0.28 for acquired-candidate ARGs; ρ = 0.07, *P* = 0.58 for total consensus ARGs). Prespecified high-risk clones are labelled in red; ST463 and ST1203 are labelled for reference.
